# Consumption of Antimicrobials in Pigs, Veal Calves, and Broilers in The Netherlands: Quantitative Results of Nationwide Collection of Data in 2011

**DOI:** 10.1371/journal.pone.0077525

**Published:** 2013-10-21

**Authors:** Marian E. H. Bos, Femke J. Taverne, Ingeborg M. van Geijlswijk, Johan W. Mouton, Dik J. Mevius, Dick J. J. Heederik, on behalf of the Netherlands Veterinary Medicines Authority (SDa)

**Affiliations:** 1 Division of Environmental Epidemiology, Institute for Risk Assessment Sciences, Utrecht University, Utrecht, The Netherlands; 2 Pharmacy, Faculty of Veterinary Medicine, Utrecht University, Utrecht, The Netherlands; 3 Department of Medical Microbiology, Radboud University Nijmegen Medical Centre, Nijmegen, The Netherlands; 4 Department of Infectious Diseases and Immunology, Faculty of Veterinary Medicine, Utrecht University, Utrecht, The Netherlands; 5 Central Veterinary Institute of Wageningen UR, Lelystad, The Netherlands; Amphia Ziekenhuis, The Netherlands

## Abstract

In 2011, Dutch animal production sectors started recording veterinary antimicrobial consumption. These data are used by the Netherlands Veterinary Medicines Authority to create transparency in and define benchmark indicators for veterinary consumption of antimicrobials. This paper presents the results of sector wide consumption of antimicrobials, in the form of prescriptions or deliveries, for all pig, veal calf, and broiler farms. Data were used to calculate animal defined daily dosages per year (ADDD/Y) per pig or veal calf farm. For broiler farms, number of animal treatment days per year was calculated. Furthermore, data were used to calculate the consumption of specific antimicrobial classes per administration route per pig or veal calf farm. The distribution of antimicrobial consumption per farm varied greatly within and between farm categories. All categories, except for rosé starter farms, showed a highly right skewed distribution with a long tail. Median ADDD/Y values varied from 1.2 ADDD/Y for rosé finisher farms to 83.2 ADDD/Y for rosé starter farms, with 28.6 ADDD/Y for white veal calf farms. Median consumption in pig farms was 9.3 ADDD/Y for production pig farms and 3.0 ADDD/Y for slaughter pig farms. Median consumption in broiler farms was 20.9 ATD/Y. Regarding specific antimicrobial classes, fluoroquinolones were mainly used on veal calf farms, but in low quantities: P75 range was 0 – 0.99 ADDD/Y, and 0 – 0.04 ADDD/Y in pig farms. The P75 range for 3^rd^/4^th^-generation cephalosporins was 0 – 0.07 ADDD/Y for veal calf farms, and 0 – 0.1 ADDD/Y for pig farms. The insights obtained from these results, and the full transparency obtained by monitoring antimicrobial consumption per farm, will help reduce antimicrobial consumption and endorse antimicrobial stewardship. The wide and skewed distribution in consumption has important practical and methodological implications for benchmarking, surveillance and future analysis of trends.

## Introduction

During the first decade of the 21^st^ century, sales of antimicrobials for veterinary consumption strongly increased in the Netherlands. [1] This is partially attributed to the ban of antimicrobial growth promoters in the European Union, which was fully effective in 2006. However, factors like intensification of animal husbandry, increase of farm size, and changing criteria for animal feed quality, due to e.g. prion prevention and economic considerations (such as more expensive compounds), have been suggested to play a role as well. [2]-[4].

More recently, antimicrobial resistance has become a growing public health problem as a result of failure of empiric treatment. [5] Prevalence and spread of antimicrobial resistant commensal and pathogenic bacteria have increased as a result of the selection for resistance because of the widespread consumption of antimicrobials. Furthermore, the same antimicrobial anatomical therapeutic chemical (ATC) classes are used in both human and veterinary medicine. [6] In the Netherlands, consumption of antimicrobials in humans is among the lowest in Europe. [7] There is also a strict infection control policy in place in most of the Dutch hospitals, intended to minimize prevalence of antimicrobial resistant pathogens in Dutch health care facilities. Although this policy did result in limitation of MRSA prevalence, extended-spectrum *β*-lactamase (ESBL)-producing Enterobacteriaceae prevalence in the Netherlands is equivalent to most other EU countries participating in EARSS. [8] Outbreaks in hospitals and other health care facilities with antimicrobial resistant pathogens are immediately dealt with, often at high costs. [9]–[11] In recent years, the emergence in livestock of antimicrobial resistant bacteria, such as livestock-associated methicillin-resistant *Staphylococcus aureus* in 2004 [12], [13] and the recent occurrence of ESBL-producing Enterobacteriaceae in the poultry production chain, led to new awareness on this issue. [14] It is still unclear to what extent antimicrobial resistant bacteria in livestock have an impact on the occurrence of antimicrobial resistant bacteria in humans, or how often the reverse is the case, but a recent study showed genetic similarities between resistant isolates found in chicken meat and humans. [15] Therefore, it is important to decrease prevalence of these bacteria in livestock, which can be achieved by, among others, decreasing antimicrobial usage in these populations. [16].

In 2010, the Dutch government demanded that veterinary consumption of antimicrobials should decrease to 50% in 2013, compared to 2009 (http://www.rijksoverheid.nl/documenten-en-publicaties/kamerstukken/2010/04/09/deskundigenberaad-rivm-en-reductie-antibioticumgebruik.html). In addition to this, the Health Council of the Netherlands advised to limit veterinary consumption of specified antimicrobials in order to preserve them for human medicine, as these are pivotal antimicrobials (e.g. fluoroquinolones and 3^rd^/4^th^-generation cephalosporins). [17] The Health Council also advised to consider limitation of all antimicrobials with selection pressure potential for ESBLs. Thus, in addition to adaptation of the national veterinary guidelines for therapy, full transparency of consumption of antimicrobials on each individual farm was required in combination with a system of benchmarking. Information on antimicrobial consumption for individual farms can identify persistent high consumers, who should give priority to consumption reduction. An independent institution, the SDa (the Netherlands Veterinary Medicines Authority), was formed in 2010 with the main purpose of creating transparency in and setting benchmark indicators for consumption of antimicrobials in livestock production, based on the consumption data as presented in the present study. Moreover, the SDa monitors, analyses, and reports data on consumption of antimicrobials on a yearly basis, thus making trends in consumption patterns in the various sectors transparent.

National overall antimicrobials sales data have been reported in the European Union for several countries for several years through the European Surveillance of Veterinary Antimicrobial Consumption (ESVAC) project of the European Medicine Agency [18]. So far, the only country registering and reporting data on sector wide veterinary consumption of antimicrobials prescribed by veterinarians, is Denmark. [19] These data are collected since 2001 on a mandatory monthly basis in a central database, VetStat [20], and reported annually. [21] In these national reports, the calculation and reporting of antimicrobial drug consumption in animal daily doses (ADDkg) was done on a national level, comparable to the human method of defined daily dosage (DDD) calculations. However, in these reports, no insight is given into the shape and width of the distribution of farm consumption. Individual farm data are available and acted upon, when considered necessary, by the Danish Veterinary and Food Administration. [22].

This paper presents the first unique results of the complete consumption of antimicrobials as registered on individual farm level, for all pig, veal calf, and broiler farms in the Netherlands in 2011.

## Materials and Methods

### Data collection

Data were collected by the respective private animal sectors and sent to the SDa after encryption of identifiers, thus ensuring the privacy of the parties concerned. In brief, the data collection process can be described as follows: each time a veterinarian prescribes and supplies medicines, information on these deliveries is entered in Practice Management Systems (PMSs) and transferred to a central database. The majority of transfers takes place through VetCIS (www.vetcis.nl), a data hub system set up by a joint collaboration of the Royal Dutch Veterinary Association (KNMvD), the main veterinary drug wholesaler in the Netherlands AUV, and the association of the veterinary pharmaceutical industry in the Netherlands (FIDIN). An external audit has been performed on data entry by the veterinarians, showing that the maximal margin of error is 10–20%. Data in VetCIS is subsequently transferred to the private sector databases. This route accounts for approximately 70% of all transferred prescriptions. Part of the data is directly transferred from the PMS to the sector databases or entered by veterinarians through internet portals of the sector systems. Farmers and veterinarians have access to the central databases through internet portals to consult prescription data and consumption of veterinary medicines. The sector data systems are part of existing integral quality assurance systems, which aim to guarantee food safety for the consumer. As a part of these systems, each farm is visited at least once a year for inspection and certification by the private certifying authorities.

Data entered per medicine delivery includes: a unique farm identifier (UFI), a unique veterinarian identifier (UVI), EAN code (unique European Article Number), number of packages supplied, animal species, animal category, and delivery date. Linked to the EAN code the following data can be collected as well: REG NL number (Dutch authorisation number for veterinary pharmaceuticals), ATCvet code, administration route, product name, and content (including unit) of packaging. Product data are derived from the so-called Branche Code Table (BCT; provided by FIDIN). Besides these data, the DD_kg_ (the Defined Dosage of medicine (g or ml) needed for the treatment of one kilogram of animal during one day) is derived from the veterinary medicine criterion (designated DG-standard) and registered in the databases. The DG-standard is a database defining standard doses for each animal species and route of administration for which an antimicrobial veterinary medicinal product is licensed in the Netherlands. In practice, DDkg is derived from the number of animal kg that, in accordance with the Specification of Product Characterization (SPC), can be treated with one ml, one gram or one piece (e.g. intramammary injector) of the specified veterinary medicinal product (VMP). It is combined with the treatment duration of this dose. For example: if the authorized dose of a VMP is 0.01 ml/kg, twice daily, this results in a treatable weight*days of (100 kg/ml*0.5 days) 50 kgdays per mL. In the case of a longacting VMP, e.g. 5 days treatment with one shot, dose again 0.01 ml/kg, this will result in (100 kg/ml*5 days) 500 kgdays per mL. The DG-standard is updated and controlled by the SDa and available on the website. The average number of animals present at the farm per year is collected on animal subcategory level, specified by age groups and farm types.

For the purpose of the first descriptive analyses, the SDa received a dataset per animal production sector containing a record for each farm with, besides the animal defined daily dosages per year (ADDD/Y; for explanation see further), UFI, UVI, data on the animal species and category, the average number of animals present on the farm during 2011 (per category and, where applicable, subcategory), empty barn period (where applicable; in days), and the period of registration of antimicrobial deliveries or prescriptions. Pig farms could be included twice in the dataset; the so-called “closed farms” breed and rear their own slaughter pigs. For the purpose of this study and benchmarking by the SDa, these farms were treated as two farm types: production and slaughter pig farms. All datasets were checked for consistency and in case of doubt, feedback was asked from the sector representatives. Where necessary, data were corrected. For veal calf farms, ADDD/Y were corrected for the periods when the barns were empty. In the pig husbandry sector, it was agreed that the - for most fattener pig husbandries consistent - factor of five days empty between two rounds of five months (3%), was not significant enough to correct for. In broilers, the empty period is already included in the variable “animal days”. Coverage of the number of farms in these databases, related to Statistics Netherlands (www.cbs.nl), was 106% for pig farms, 110% for veal calf farms, and 122% for broiler farms. Discrepancies may be caused by differences in the definition of “farm” and timing of registration of the number of farms. On individual farms, the total of antimicrobial deliveries or prescriptions could be below 0 ADDD/Y. Reversed entries are made when unused antimicrobials are returned to the veterinarian or when a wrong entry was made.

For analysis of consumption of specific antimicrobial classes for each route of administration, the SDa also received prescription data for the veal calf and pig sectors. For broiler farms, these data were not yet completely available in sufficient detail and are not presented.

In these datasets, each veterinary drug delivery on a farm is recorded, containing, amongst others, identifiers, EAN code, number of packages, and delivery date. For calculation of ADDD/Y, prescription data was linked with the other datasets to add the number of animals present on the farm.

We will further use the term “antimicrobial consumption” as a synonym for “antimicrobial delivery”, as we assume that each delivery is either consumed by animals, or returned to the veterinarian and a reversed entry is made for this delivery.

### Calculation of ADDD/Y and ATD/Y

The sectors calculated the consumption of antimicrobials per farm as animal defined daily dosages per year (ADDD/Y); apart from the denominator this is similar to the standard unit for consumption of antimicrobials in humans (DDD/1000 days). An ADDD/Y of 1 means that the average animal in the population was exposed to an antimicrobial for one day per year. This measure is similar to that proposed by ESVAC. [23].

To calculate ADDD/Y, two variables are needed. First, the total animal mass in kg that can be treated for one day with the amount of antimicrobials supplied to the farmer (treatable weight*days), which is derived from the DG-standard. Second, the mean total weight (kg) of animals present on the farm during 2011 (animal weight), which can be calculated according to standardised mean animal weights (determined per production type and animal category, see Table 1). By dividing these numbers, the number of animal daily dosages per year is obtained.

**Table 1:** The applied standardised mean animal weight and treatment weight per animal category and age category (adapted from http://www.autoriteitdiergeneesmiddelen.nl/Userfiles/rapportage--sda-expertpanel-dataanalyse-2011-en-benchmarkindicatoren-2012.pdf).

Animal species	Farm category	Age	Standardised mean weight
Veal calf	White	0 – 222 days	160 kg
	Rosé starter	0 – 98 days	77.5 kg
	Rosé finisher	98 – 256 days	232.5 kg
Pig	Production with sows and piglets	Combination of ages	303.8 kg*
	Slaughter	74 – 191 days	70 kg

* Combination of 1 sow (of 220 kg) + 5.5 piglets (of 12.5 kg each) + 0.14 gilts (of 107.5 kg each).

For broilers, ADDD/Y could not yet be determined, because these data did not include the variables needed to calculate treatable kilograms. Instead, animal treatment days per year (ATD/Y) were calculated. One difference between ADDD/Y and ATD/Y is that the number of treatment days is based on prescriptions by a veterinarian for a specific age category, independent of dosage or duration of effectiveness. Another difference is that the calculation of ATD/Y does not include a standardised mean weight for the animals. For calculation of ATD/Y, the numerator was the summation of the number of treatment days for all broilers present during the year (eg., when 100 animals were each treated for 5 days, the number of treatment days would be 100*5 = 500). The denominator was denoted by the number of animal days; i.e. the sum of the number of birds present per day for the year (eg., when during a whole year on average 100 animals are present, the number of animal days is 100*365 = 36500). By dividing these numbers and multiplying by 365, the number of ATD/Y was obtained: the number of days antimicrobials were administered to broilers on a farm per year (in our example: (500/36500)*365 = 5).

### Statistical analysis

Descriptive statistics were obtained using the MEANS and FREQ procedures in SAS® software version 9.2 (SAS Institute Inc., Cary, NC, USA). Statistical analyses were performed on the data by means of the REG and TTEST procedures. We applied univariate models with ADDD/Y as the dependent variable to test for the influence of farm characteristics (such as farm size) on antimicrobial use. R^©^ open source software version 2.15.2 (R Foundation for Statistical Computing, Vienna, Austria) was used for creating (density) plots.

## Results

### Farm descriptives

**Figure 1:**
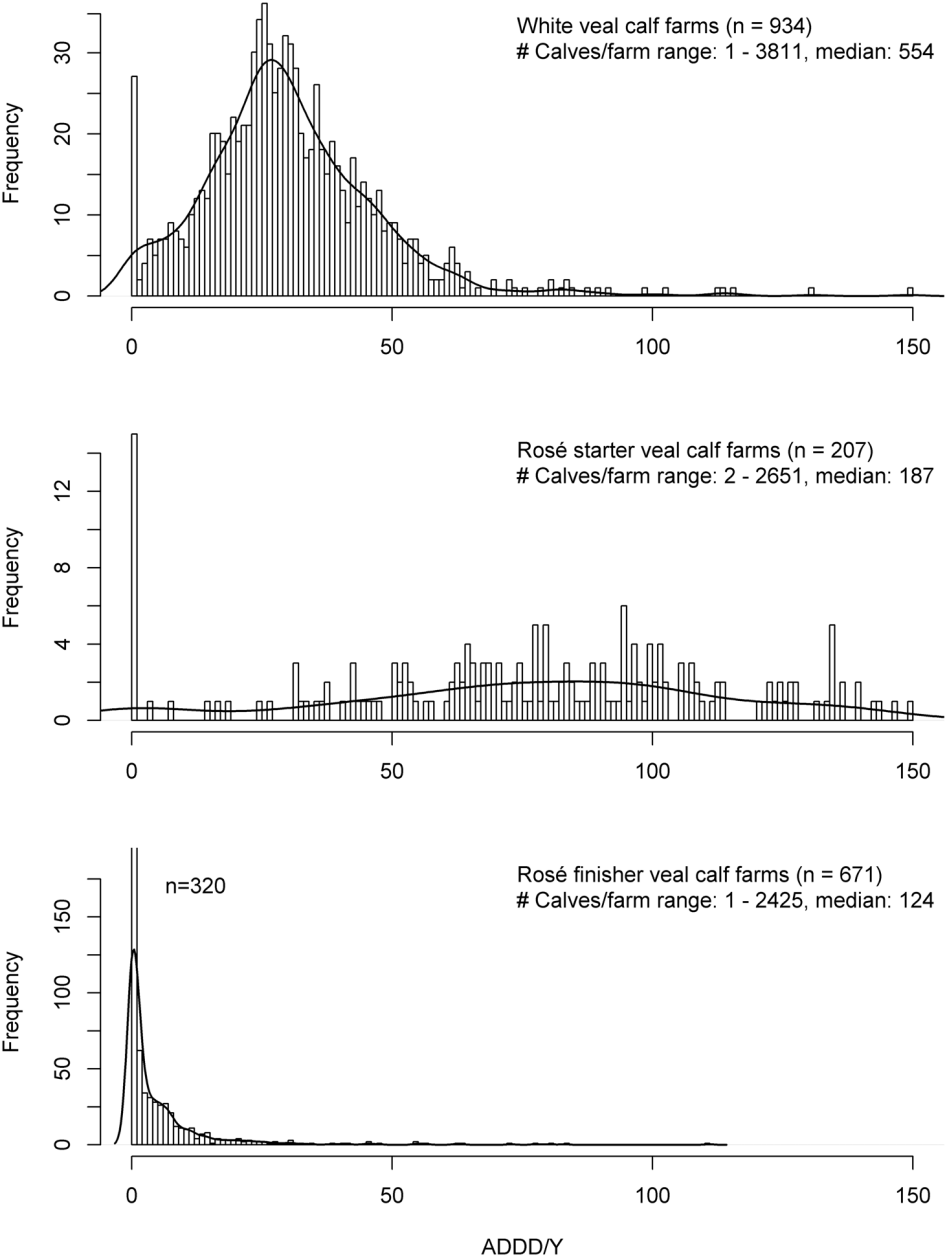
Frequency distribution of ADDD/Y per veal calf farm ranging from 0 to 150. The line demonstrates a density plot based on the histogram. Number of farms with ADDD/Y>150: 27 (white: 7, rosé start: 20) and number of farms with ADDD/Y<0: 4 (white: 3, rosé finisher: 1).

The majority of the 2125 veal calf farms in the data consisted of white veal calf farms (n = 934, 44%), followed by rosé finisher farms (n = 671, 32%), rosé starter farms (n = 207, 10%), and farms on which both rosé starter and finisher calves were present, or a combination of white and rosé calves (n = 313, 15%) (figure 1). There were 14 combination farms in the data with no animals present; these have been excluded from the results. Registered farm sizes were diverse, ranging from one animal present in 2011 to over 3000 animals, with mean numbers of 606 (white veal), 285 (rosé starter), 210 (rosé finisher), and 437 (combination) animals per farm. There were 2528 production farms with sows and piglets in the data, and 5531 farms where slaughter pigs were kept. Two production farms had no animals in 2011; these have been excluded from further results. Again, registered pig farm sizes varied greatly, ranging from 2 sows to over 4000 for production farms, and from 2 slaughter pigs to 13000 for slaughter pig farms. Mean number of sows for production farms was 382, and for slaughter pig farms 1092 animals. The 732 broiler farms ranged from 620 to 434106 (mean: 67745) broilers.

### Antimicrobial consumption

**Figure 2:**
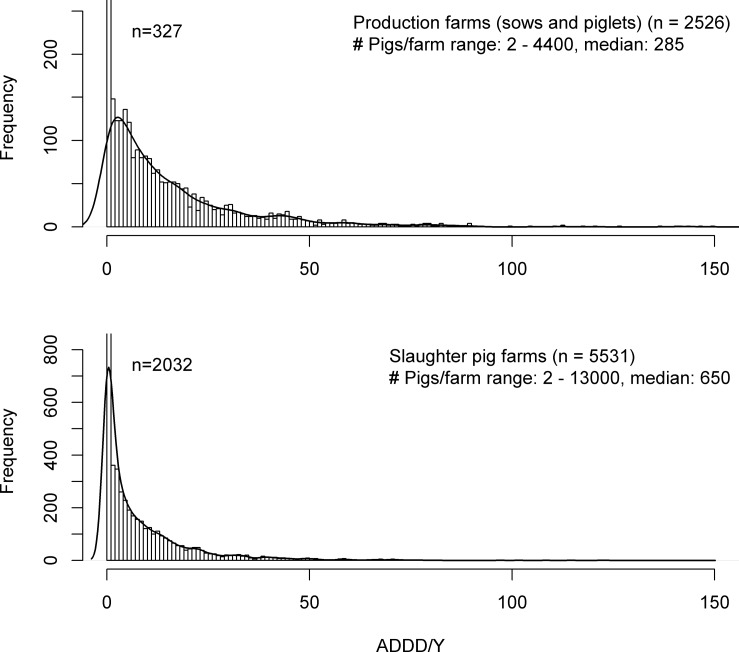
Frequency distribution of ADDD/Y per pig farm ranging from 0 to 150. The line demonstrates a density plot based on the histogram. Number of farms with ADDD/Y>150: 15 (sows: 3, finishers: 12) and number of farms with ADDD/Y<0: 1 (finisher: 1).

**Figure 3:**
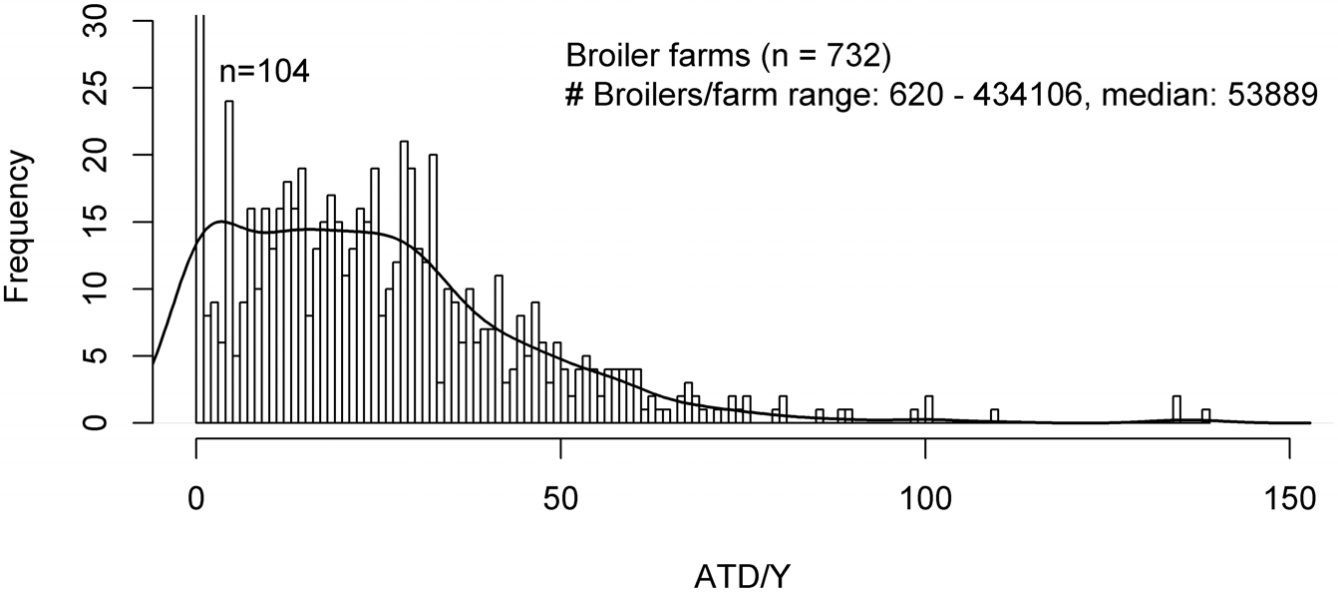
Frequency distribution of ATD/Y per broiler farm ranging from 0 to 150. The line demonstrates a density plot based on the histogram. Number of farms with ATD/Y>150: 0 and number of farms with ATD/Y<0: 0.

The distributions of ADDD/Y and ATD/Y per farm are shown in Figures 1, 2, 3 for the various livestock categories. The distributions for white and rosé starter farms showed a large spread in ADDD/Y values. With exception of rosé starter farms, all distributions were highly skewed to the right and showed a long tail.

Table 2 shows the arithmetic and geometric means and standard deviations, and the median and 75^th^ percentile (P75) for ADDD/Y for veal calf and pig farms, and ATD/Y for broiler farms. The highest arithmetic mean and median ADDD/Y were found in rosé starter farms (arithmetic mean/median ADDD/Y  =  105.4/83.2, P75  =  110.0, 90^th^ percentile (P90)  =  149.5), and the lowest arithmetic mean and median ADDD/Y in rosé finisher farms (arithmetic mean/median ADDD/Y  =  5.2/1.2, P75  =  6.0, P90  =  13.1). White veal calf farms had an intermediate consumption of antimicrobials (arithmetic mean/median ADDD/Y  =  35.6/28.6, P75  =  38.9, P90  =  50.7). ADDD/Y values were not calculated for combination farms, because of their complex nature with regards to the categories of calves present.

**Table 2:** Arithmetic mean and standard deviation, and geometric mean and standard deviation of ADDD/Y for veal calf and pig farms, and of ATD/Y for broiler farms.

Animal species	Farm category	Arithmetic mean	Arithmetic standard deviation	Median	P75	Geometric mean*	Geometric standard deviation*
Veal calf	All active farms**	32.2	125.4	19.7	35.6	8.1	9.0
	White	35.6	111.5	28.6	38.9	23.2	3.1
	Rosé starter	105.4	159.1	83.2	110.0	52.5	6.5
	Rosé finisher	5.2	10.9	1.2	6.0	1.1	7.2
Pig	Production with sows and piglets	16.9	58.9	9.3	20.8	6.3	5.8
	Slaughter	9.6	48.0	3.0	10.8	1.8	8.4
Poultry	Broiler	23.8	20.6	20.9	34.1	9.9	7.5

* In order to calculate the natural log, ADDD/Y ≤ 0 was set at 0.1.

** Farms where no animals were present were excluded, 2111 farms are included in this category.

In the pig sector, the arithmetic mean/median ADDD/Y were 16.9/9.3 (P75  =  20.8, P90  =  40.6) and 9.6/3.0 (P75  =  10.8, P90  =  22.5) for production and slaughter pig farms, respectively. Broiler farms had an arithmetic mean/median ADT/Y of 23.8/20.9 (P75  =  34.1, P90  =  50.0).

No univariate significant association was found between consumption of antimicrobials and the number of veal calves present as a continuous variable (p-values > 0.1), or as a categorical variable (smaller or larger than the median, p-values > 0.09). However, for the number of pigs present on the farm, a positive significant univariate association with consumption of antimicrobials was found (production farms: β = 0.012, 95% Confidence Interval (CI): 0.006 – 0.019, p < 0.01; slaughter pig farms: β = 0.0017, 95% CI: 0.0007 – 0.003, p < 0.01). A similar result was seen for production pig farms when analysing number of pigs as a categorical variable depending on the median. For the number of broilers present on the farm, a significant, albeit small, positive association with consumption of antimicrobials was found as well (β = 0.00006, 95% CI: 0.00004 – 0.00009, p < 0.01).

**Figure 4:**
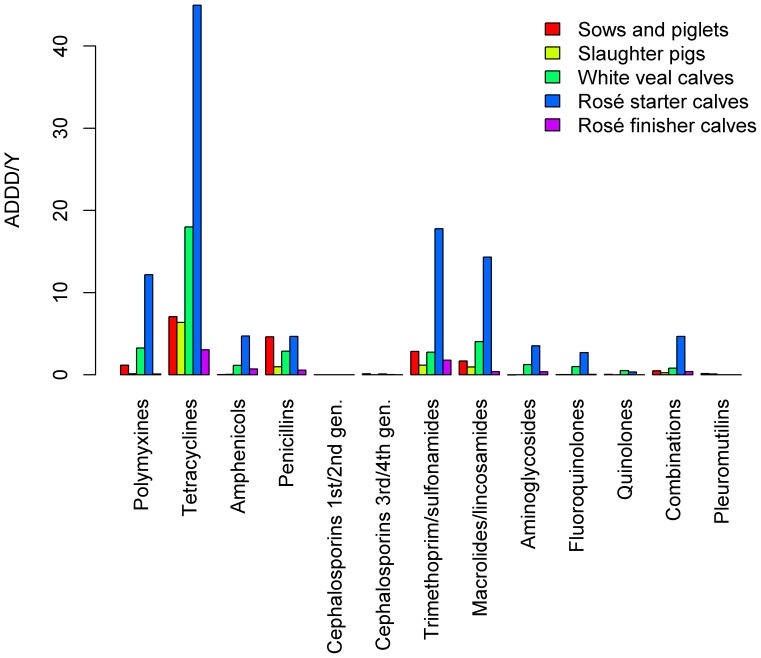
Mean ADDD/Y per farm per animal sector, given for 13 ATCvet classes.

In figure 4 an overview is given of the mean consumption of antimicrobials per farm category in the pig and veal calf sectors, for specified ATCvet groups. The figure shows that tetracyclines were overall the most used antimicrobial group, followed by trimethoprim/sulfonamides and macrolides/lincosamides.

On 39% of 934 white veal calf farms, 3^rd^/4^th^-generation cephalosporins were consumed in 2011, with a P75 of 0.07 ADDD/Y. Fluoroquinolones were administered orally on 59% of the white veal calf farms (P75  =  0.32 ADDD/Y), and parenterally on 87% of these farms (P75  =  0.34 ADDD/Y). Rosé starter and finisher calf farms hardly used 3^rd^/4^th^-generation cephalosporins (13%/10% of 207 starter and 665 finisher farms, respectively, with P75  =  0 ADDD/Y). Fluoroquinolones were administered on most rosé starter farms in 2011 (40% of the farms orally: P75  =  0.50 ADDD/Y, and 75% parenterally: P75  =  0.99 ADDD/Y), but hardly on rosé finisher farms (2% of the farms orally: P75  =  0 ADDD/Y, and 24% parenterally: P75  =  0 ADDD/Y).

Consumption of these antimicrobials was low on pig farms. On 22% (P75  =  0 ADDD/Y) and 4% (P75  =  0 ADDD/Y) of 2494 production and 5441 slaughter pig farms 3^rd^/4^th^-generation cephalosporins were administered, respectively. Fluoroquinolones were administered orally on 4% (P75  =  0 ADDD/Y) and 0.1% (P75  =  0 ADDD/Y) of the production and slaughter pig farms, respectively. Parenterally, these antimicrobials were administrated on 36% (P75  =  0.04 ADDD/Y) and 8% (P75  =  0 ADDD/Y) of the pig production and slaughter pig farms.

## Discussion

The unique data presented in this study represent the first time that consumption of antimicrobials, in the form of prescriptions and/or deliveries on the farm, is available on this level for each pig, veal calf, and broiler farm in the Netherlands. The data show that large differences exist between farms within the different categories regarding consumption of antimicrobials. Consumption also greatly varies between farm categories. In most categories analysed here, the majority of farms have low consumption of antimicrobials. However, a minority of farms have a very high ADDD/Y. The insights obtained from these data aid the SDa with their aim to define benchmark criteria for antimicrobial consumption, which should ultimately lead to reduction in consumption.

ADDD/Y is a measure for the number of days on a farm antimicrobials have been administered. It is important to realize that this does not mean that each and every animal has received that calculated ADDD/Y, but that it in fact is a measure per animal position available on the farm. ADDD/Y is also based on standard doses as determined by the SDa, which in general is the mean dose according to the Specification of Product Characterization (SPC) of the specific veterinary medicinal product. In reality, under- or overdosing may occur frequently. [24] Other factors influencing the calculated ADDD/Y include in the numerator long acting products and in the denominator the selection of the average weight (such as the combination of sows and piglets). For long acting products a treatment duration factor is added to the ADDD calculation (for instance a single shot of a product acting for 5 days results in 5 times the number of ADDD compared to a single shot of a direct acting drug).

On mixed farms, such as combination veal calf farms (with both rosé and white veal calves present) and “closed” pig farms (with both sows and piglets and slaughter pigs), it is possible that antimicrobials have not been registered for the correct animal category. This may lead to misclassification and a biased ADDD/Y per farm. However, this effect is expected to be small over a farm category. In the current analyses, closed pig farms have been included as two farms, and combination veal farms have been ignored. In future analyses, these farms will be considered as distinct farm categories.

Clear differences exist in antimicrobial consumption between animal sectors and farm categories, but these are difficult to compare due to differences in farm management. The veal calf sector has a higher consumption, which might be a consequence of the way calves are collected. Veal calf farms receive calves from dairy farms from the Netherlands (approximately 50%) and countries throughout Europe (predominantly Germany). These calves are a product of the dairy industry. [25] Sober calves arrive once or twice a year (usually an all-in, all-out system is applied), after transport often creating an optimal atmosphere for pathogen transmission. Frequently, calves receive antimicrobial treatments in the first few weeks after arrival, which means that farms with two production cycles in a year, likely will have a higher ADDD/Y then farms with only one starting phase of a production cycle in that particular year. [24]–[26].

Dutch pig farms have more closed production systems than veal calf farms, or have animal supply from limited sources. In broilers, infection control is well possible by hygienic measures between production rounds. However, broiler production in the Netherlands is not integrated in production chains that include breeders, hatcheries and broiler farms. As a result, infection control is also not optimally integrated along these production chains. Currently, best practices to control infectious diseases and the health status of animals is a priority in the Netherlands, both within animal production sectors and within the veterinary society.

Comparisons between animal sectors or farm categories should be made with care because of the influence of animal weight on the calculated ADDD/Y. Calculation of ADDD/Y is based on a standardised average weight for animals. For adult animals, e.g. sows, this weight will be steady over the course of a year, but for fattener animals, e.g. white veal calves and slaughter pigs, this weight will strongly increase during the production cycle. Recent studies showed that the majority of antimicrobial use in pigs is administered before they are 10 weeks of age, i.e. at lower weight, which is comparable to treatments in veal calves. [26], [27] For the category of white veal calves and slaughter pigs, depending on the exact timing of the treatment over- but more likely underestimation of the ADDD/Y can occur. Use of actual weights during treatment could improve the ADDD/Y estimate but requires a more detailed and accurate registration. [24].

To date, Denmark is the only other country publishing antimicrobial consumption figures annually, based on prescriptions by veterinarians, for all farms in an animal sector. [21], [28] In the DANMAP reports, antimicrobial drug consumption is presented as animal daily doses (ADDkg), related to total biomass-year-at-risk, kg-meat-produced, or number of animals produced. ADDkg is given for each age-group and species, e.g. piglets, weaners, fattening pigs or sows, but no mean farm ADD is reported in the reports. [19] Denmark does, however, calculate farm level ADDs for their “yellow card” system, which is designed to control veterinary antimicrobial consumption. [22] In this system, pig farms are given a “yellow card” when they consume more than twice the average consumption. Farms with a “yellow card” have to implement antimicrobial restrictive measures.

The European Medicines Agency is currently considering which technical units of measurements and indicators should be used for future collection of and reporting on national veterinary antimicrobial drug consumption, as part of the European Surveillance of Veterinary Antimicrobial Consumption project. [23] Although this will provide valuable information on national level, neither the proposed ESVAC indicators for the near future nor the DANMAP indicators as reported up till now, consider the practical and methodological implications of the wide distribution of antimicrobial consumption on farms, as shown in this paper. Our farm level data, reporting on all individual farms nationwide, create a breakthrough in analytical possibilities and benchmarking options, as we clearly demonstrate that normal distribution statistics are not appropriate for describing or analysing antimicrobial consumption, and that differences in consumption of antimicrobial agents amount to one, sometimes two orders of magnitude between farms.

In previous years, veterinary consumption of antimicrobials in the Netherlands was reported for a selection of farms (based on stratified sampling) and in terms of total national sales data. [1], [29] Bondt *et al.* used national sales data to compare veterinary antimicrobial exposure between Denmark and the Netherlands, demonstrating that data on consumption per animal species is a necessity to be able to adequately compare countries. [30] When comparing our data (table 2) with those of the MARAN-report on 2011, we demonstrate that outcomes based on a sample of farms may give a biased estimate of antimicrobial consumption. [31] The MARAN-report shows consistently lower results when comparing to the arithmetic means in table 2. The mean arithmetic consumption in daily doses per animal year (DD/AY) reported by MARAN is 13 (95% CI: 10 – 16) for pig production farms, 8 (95% CI: 5 – 11) for slaughter pig farms, 25 (95% CI: 23 – 26) for veal calf farms, and 16 (95% CI: 12 – 21) for broiler farms. However, when we compare the MARAN results with our calculated geometric means, our results are much lower. This shows that the long right tail in the distributions strongly influences the average consumption in a farm category, and emphasizes the need for reporting distributions of consumption on farm level. The use of a disproportional stratified random sampling design for the MARAN-report probably also affected the outcomes, where the weighted results of a selection of farms are extrapolated to the whole sector.

So far, the reason for the large and right skewed between-farm spread in consumption of antimicrobials is unknown. In veal calf farms the number of production cycles started in 2011, may have contributed to the skewedness of the distribution, as younger calves are more susceptible to infections and therefore will receive more treatments with antimicrobials. This may lead to a relatively higher consumption in farms with more production cycles in a year. A similar wide between-farm spread has been shown by other studies. [2], [32] For slaughter pig farms, farm system (farrow-to-finish or specialized slaughter pig farms) and number of pigs were shown to influence consumption of antimicrobials, and for sow farms this was farm system (specialized sow farm), number of sows and regional population density. [2] We analysed the influence of farm size on consumption of antimicrobials, as earlier reports on Dutch sentinel farms found an association in pig production (but not in poultry). [3] In our data, this association was found to be significant for the pig and broiler farms, but in univariate analyses only, therefore not accounting for the influence of other factors. The influence of farm size needs to be studied in more detail.

For 2012, the SDa have defined benchmark action criteria for antimicrobial consumption. Because of the wide distributions, the initial aim is to limit the tail of the distribution, and benchmarks focus at the 75^th^ percentile of the consumption in 2011. Farms that exceed these benchmarks are obliged to promptly undertake measures to decrease their consumption of antimicrobials. Moreover, in general median consumption in 2011 minus 20% was defined as the upper limit for the target benchmark criterion for appropriate antimicrobial consumption. This target will be re-evaluated in 2015. The benchmark criterion for action will annually be re-evaluated, because it is anticipated that removing the long tail in the population will have a major impact on the average consumption in a sector. When all farms above the action benchmark criterion reduce their antimicrobial consumption to the upper limit of the target benchmark criterion, the overall decrease of antimicrobial consumption in each of the farm categories will be substantial. Early 2013, data for 2012 have become available that will allow the evaluation of the achieved reduction and persistent high users. Data on broiler farms will be provided similar to the other animal sectors, facilitating calculation of ADDD/Y for broiler farms instead of ATD/Y. In addition, antimicrobial prescription behaviour will also be monitored on the level of veterinarians.

In conclusion, the analysis of unique data provided to the SDa by the animal sectors in the Netherlands, shows that consumption of antimicrobials varies strongly between animal production sectors and farm categories, and also within farm categories. The wide and skewed distribution in consumption has important methodological implications for benchmarking, surveillance, and future analyses of trends over the years within farms, within animal sectors, and between animal sectors. The full transparency obtained on antimicrobial consumption per farm, as shown in the data collected for the SDa, enables targeted measures to reduce and improve the quality (in terms of very restricted antimicrobial use of specified groups like fluoroquinolones and cephalosporins) of antimicrobial consumption, and serves as a tool for both farmers and veterinarians. These will include measures to improve health status and control of infectious diseases on those farms where consumption of antimicrobials is (consistently) highest. This should result in optimal reduction of antibiotic consumption combined with improved health control. In the following years, the SDa will report on within-(sub)sector trends, expanding to other animal sectors, as well as on within-farm trends and trends on prescriptions by veterinarians.
